# Occurrence of Aflatoxin B_1_, deoxynivalenol and zearalenone in feeds in China during 2018–2020

**DOI:** 10.1186/s40104-021-00603-0

**Published:** 2021-07-10

**Authors:** Ling Zhao, Lei Zhang, Zijian Xu, Xingda Liu, Liyuan Chen, Jiefan Dai, Niel Alexander Karrow, Lvhui Sun

**Affiliations:** 1 Department of Animal Nutrition and Feed Science, College of Animal Science and Technology, Huazhong Agricultural University, Wuhan, 430070 Hubei China; 2 Guilin Li Yuan Grain and Oil Food Group Co., Ltd, Guilin, 541001 Guangxi China; 3 Jiangsu Aomai Bio-Technology Co., Ltd, Nanjing, 211226 China; 4 Department of Agriculture of Sichuan Province, Chengdu, 610041 China; 5 Department of Animal Biosciences, University of Guelph, N1G2W1, Guelph, Canada

**Keywords:** Aflatoxin B_1_, China, Deoxynivalenol, Feeds, Zearalenone

## Abstract

**Background:**

The current study was conducted to investigate the individual and combined occurrence of aflatoxin B_1_ (AFB_1_), deoxynivalenol (DON) and zearalenone (ZEN) in feeds from various Provinces of China during 2018 to 2020. A total of 3,507 feed samples, including 2,090 feed ingredients and 1,417 complete feed samples, were collected from different areas of China for mycotoxins analysis.

**Results:**

The individual contamination of AFB_1_, DON and ZEN were present in more than 81.9%, 96.4% and 96.9% of feed samples, respectively, with average concentration ranges of AFB_1_ between 1.2–27.4 μg/kg, DON between 458.0–1,925.4 μg/kg and ZEN between 48.1–326.8 μg/kg. Notably, 0.9%, 0.5% and 0.1% of feed ingredients, and 1.2–12.8%, 0.9–2.9% and 0–8.9% of complete feeds for pigs, poultry and ruminants with AFB_1_, ZEN and DON that exceeded China’s safety standards, respectively. Moreover, more than 81.5% of feed ingredients and 95.7% of complete feeds were co-contaminated with various combinations of these mycotoxins.

**Conclusion:**

This study indicates that the feeds in China were universally contaminated with AFB_1_, DON and ZEN during the past 3 years. These findings highlight the significance of monitoring mycotoxin contaminant levels in the domestic animal feed, and the importance of carrying out feed administration and remediation strategies for mycotoxin control.

## Introduction

Mycotoxins are naturally toxic secondary metabolites produced by various molds, including *Aspergillus*, *Alternaria*, *Claviceps*, *Fusarium* and *Penicillium* [1]. More than 500 mycotoxins have been identified to date [2]. Aflatoxin B_1_ (AFB_1_), deoxynivalenol (DON) and zearalenone (ZEN) are recognized as the primary toxins occurring in agricultural commodities, such as maize, peas, peanuts, wheat, barley, millet, nuts, oily feedstuffs, forage, and their by-products [3–5]. Mainly generated by *Aspergillus*, AFB_1_ is the most lethal toxin, exhibiting hepatotoxic, carcinogenic, mutagenic, and teratogenic properties in animals and humans [6–8]. Both DON and ZEN are primarily generated by *Fusarium* molds. DON is a type B trichothecene, which can cause anorexia, emesis, and impairs intestinal and immune function by inhibiting nucleic acid and protein synthesis in livestock [5, 9–11], while ZEN is an estrogenic mycotoxin, that can induce reproductive and fertility disorders by competing with 17 β-estradiol for estrogen receptor binding [11–14].

Since mycotoxins can affect animal production, as well as product quality and safety, safety standards for mycotoxins in feedstuffs and feed have been established world-wide. For example, the European Commission set limitations of AFB_1_, DON, and ZEN at 5–20 μg/kg, 900 μg/kg, and 250 μg/kg, respectively, for all kinds of feedstuffs and feed [15, 16]. In 2017, China’s General Administration of Quality Supervision, Inspection and Quarantine released the latest version of safety standards (GB 13078–2017) for AFB_1_, DON, and ZEN; which are 10–20 μg/kg, 1,000–5,000 μg/kg, and 100–250 μg/kg, respectively, for feedstuffs and complete feeds (Table 1) [17].

**Table 1:** China’s feed safety standards for Aflatoxin B_1_, deoxynivalenol and zearalenone

Feeds	Maximum limit, μg/kg
Aflatxoin B_1_	
Corn by-products and peanut cake	50
Vegetable oil (except corn oil and peanut oil)	10
Corn oil and peanut oil	20
Other plant feed ingredients	30
Complete feeds for young pigs and poultries	10
Growing complete feeds for boilers and meat duck and laying ducks	15
Concentrate supplement for calf, lamb and lactation period	20
Concentrate supplement for lactation period	10
Concentrate supplement for others	30
Other complete feeds	20
Deoxynivalenol	
Plant feed ingredients	5,000
Concentrate supplement for calf, lamb and lactation period	1,000
Concentrate supplement for others	3,000
Complete feeds for pigs	1,000
Other complete feeds	3,000
Zearalenone	
Corn and its by-products (except corn bran and corn steep powder)	500
Corn bran and corn steep powder	1,500
Other plant feed ingredients	1,000
Concentrate supplement for calf, lamb and lactation period	500
Complete feed for young pigs	150
Complete feed for young gilts	100
Other complete feeds for pigs	250
Other complete feeds	500

Global climate change is increasing crop susceptibility to fungal infection, which is further causing increased mycotoxin contamination of staple cereals [18, 19]. China’s agriculture sector is highly susceptible to mycotoxin contamination in several climatic regions across the country; for example, the warm or humid conditions of the Yangtze, Yellow River basins and northeast region and their numerous rainfall events, are favorable for mold growth and mycotoxin production in crops [20–22]. Therefore, monitoring mycotoxin concentrations in the feedstuffs and complete feeds from these and other regions across China is essential to prevent farm animal exposure to mycotoxins and to ensure feed and food safety. Thus, the current study was conducted in order to investigate the individual and combined contamination of AFB_1_, DON and ZEN in feedstuffs and complete feeds collected from different regions of China.

## Materials and methods

### Samples collection and preparation

A total of 3,507 feeds samples were collected during 2018 to 2020 from either feed companies or livestock farms in different regions of China. There were 2,090 feedstuff samples including 699 corn, 127 dried distillers grains with soluble, 61 corn germ meal, 68 corn bran, 26 corn gluten meal, 171 wheat, 108 wheat middling, 275 wheat bran, 17 wheat flour, 177 soybean meal, 24 soybean bran, 33 rapeseed meal, 41 peanut meal, 79 fish meal, 125 grass grain, 41 unite bran, 18 rice bran, along with 1,417 complete feed samples including 620 pig feed, 572 poultry feed and 225 ruminant feed. These feed samples were primarily collected from the provinces of Anhui, Beijing, Chongqing, Fujian, Guangdong, Guangxi, Gansu, Henan, Hebei, Hunan, Hubei, Heilongjiang, Inner Mongolia, Jiangsu, Jiangxi, Jilin, Liaoning, Ningxia, Shandong, Sichuan, Shanxi, and Zhejiang. Since few feed samples with insufficient quantity, 3,500, 3,507 and 3,499 samples were analyzed for AFB_1_, DON and ZEN, respectively. The feed samples were stored in sealing bags at − 20 °C before analysis.

### Extraction of mycotoxins from samples

AFB_1_, DON and ZEN were extracted from the feed samples as previously described [4, 22, 23]. Briefly, 25 g of the mashed feed samples were mixed with a 100 mL solution of methanol: water (80, 20, *v*/*v*), methanol: water (60, 40, *v*/*v*) and acetonitrile: water (84, 16, *v*/*v*) for AFB_1_, DON and ZEN isolation, respectively. The samples were blended using a commercial blender at high speed for 3 min and filtered using a Mycosep® #226 column (Romer Labs. Inc., Singapore). The solvent extracts were diluted with phosphate-buffered saline solution (PBS, pH 7.4), then washed with PBS and methanol-water solution through immunoaffinity columns; AokinImmunoClean CF AFLA and CF DON (Aokin AG, Germany) for AFB_1_ and DON, respectively, and ZearaStar (Romer Labs, Austria) for ZEN. Finally, the mycotoxins were eluted from the columns using methanol, and concentrated to dryness under a nitrogen air steam. The mycotoxin residues were then re-dissolved in a mobile phase described below, filtered through a Millex PTFE 0.22 μm filter (Merck, Tianjin, China), and analyzed by high-performance liquid chromatography (HPLC).

### HPLC analysis

The mycotoxins were quantified followed the national standard methods as previously described [4, 22–25]. Briefly, AFB_1_ concentrations were measured with a reverse-phase HPLC/fluorescence detection system (Agilent 1260, Agilent Technologies, Waldbronn, Germany) with a 360 nm excitation and 440 nm emission fluorescence detector. A C_18_ column (4.6 mm × 250 mm, 5 μm, Dikma, Shanghai, China) was employed with the limit of detection (LOD) and quantification (LOQ) set at 0.5 μg/kg and 1.5 μg/kg, respectively. A mobile phase of methanol: water: acetonitrile (30, 60, 10, *v*/*v*/*v*) was used for AFB_1_ analysis at a flow rate of 1 mL/min, and the column temperature was set at 30 °C. DON and ZEN concentrations were measured using a Shimadzu LC-20A binary gradient liquid chromatograph (Shimadzu Europa GmbH, Duisburg, Germany) equipped with a C_18_ (4.6 mm × 150 mm, 5 μm) reverse-phase column (ZORBAX Eclipse XDB-C18, Agilent Technologies, Waldbronn, Germany). The mobile phase for DON analysis consisted of methanol: water solution (20, 80, *v*/*v*) at a flow rate of 0.8 mL/min under UV light at a wavelength of 218 nm [24], and the LOD and LOQ for DON were 100 μg/kg and 260 μg/kg, respectively. A mobile phase of methanol: water: acetonitrile (8, 46, 46, *v*/*v*/*v*) was used for ZEN analysis at a flow rate of 1 mL/min under 274 nm excitation and 440 nm emission wavelengths [25]; the LOD and LOQ for ZEN were 10 μg/kg and 24 μg/kg, respectively. The blank samples are the solvents that were used to dissolve standard samples before HPLC analysis. LOD and LOQ correspond to the amount of analyte for which the signal-to-noise ratio is equal to 3 and 10 [26, 27], respectively, with a minor adjustment according to our previous study [23].

### Statistical analysis

All the data were analyzed by the Microsoft Excel 2003 (Microsoft Corporation, Redmond, USA) and expressed as means, median, maximum, or percentages.

## Results

### Occurrence of AFB_1_ in feeds

A total of 3,500 feed samples, including 2,083 feedstuff and 1,417 complete feeds, were collected between 2018 and 2020 for analysis of AFB_1_ (Table 2). AFB_1_ was detected in 81.9–100% of feedstuff and complete feeds, with the average levels ranging from 1.2–27.4 μg/kg. The highest median concentration of AFB_1_ was 32.0 μg/kg in peanut meal from the 2019 harvest, followed by 15.6 μg/kg in corn bran from 2020 and 10.8 μg/kg in complete ruminant feed from the 2019 harvest. The maximum levels of AFB_1_ were 221 μg/kg in corn harvested in both 2018 and 2019, followed by 77.5 μg/kg in both ruminant complete feed from 2018 and wheat middling from 2019, and 68.7 μg/kg in corn bran from 2018. Only 18 raw feed ingredient samples, which account for 0.9% of all the analyzed feedstuffs, were contaminated with AFB_1_ at concentrations over the Chinese safety standard concentration of 250 μg/kg (Table 1). Notably, 9 samples of complete pig feed, 7 samples of complete poultry feed and 29 samples of complete ruminant feed, which account for 1.5%, 1.2% and 12.8% of all the analyzed samples were contaminated with AFB_1_ at levels exceeding Chinese safety standard concentrations (Table 1).

**Table 2:** Aflatxoin B_1_ concentrations in feeds^a^

Item	Year	NO. of samples	Positive samples, μg/kg				Numbers of samples in the range, μg/kg					The rate of over standard, %
			%	Mean	Medain	Maximum	< 0.5	0.5–10	10–30	30–50	> 50	
Corn	2018	229	95.6	4.4	1.9	221.0	10	210	5	0	4	1.7
	2019	249	81.9	3.9	2.1	221.0	45	197	6	0	1	0.4
	2020	215	98.1	3.7	3.4	11.8	4	208	3	0	0	0
Dried distillers grains with soluble	2018	82	100	7.9	5.2	45.8	0	55	26	1	0	0
	2019	22	100	7.8	6.2	17.9	0	14	8	0	0	0
	2020	23	95.7	4.9	4.2	11.2	1	21	1	0	0	0
Corn germ meal	2018	28	100	7.0	3.5	40.2	0	24	2	2	0	0
	2019	23	100	7.1	3.3	22.4	0	16	7	0	0	0
	2020	10	100	7.5	4.6	30.8	0	8	1	1	0	0
Corn bran	2018	33	100	9.4	3.6	68.7	0	26	4	2	1	3.0
	2019	19	100	4.3	3.3	17.9	0	18	1	0	0	0
	2020	16	100	8.6	7.3	29.3	0	11	5	0	0	0
Corn gluten meal	2018	21	100	5.0	2.5	27.9	0	19	2	0	0	0
	2019	4	100	7.8	3.9	21.1	0	3	1	0	0	0
	2020	1	100	15.6	15.6	15.6	0	0	1	0	0	0
Wheat	2018	110	99.1	2.8	2.7	7.0	1	109	0	0	0	0
	2019	34	97.1	3.4	3.2	8.2	1	33	0	0	0	0
	2020	27	100	3.6	2.9	11.4	0	26	1	0	0	0
Wheat middling	2018	34	100	2.7	2.8	4.1	0	34	0	0	0	0
	2019	46	100	6.5	2.8	77.5	0	43	1	0	2	4.3
	2020	28	100	3.1	3.1	6.4	0	28	0	0	0	0
Wheat bran	2018	148	100	6.1	3.7	57.4	0	125	20	2	1	2.0
	2019	86	100	4.3	3.4	12.3	0	81	5	0	0	0
	2020	41	100	4.5	3.5	25.5	0	38	3	0	0	0
Soybean meal	2018	118	100	2.3	2.1	5.7	0	118	0	0	0	0
	2019	23	100	2.8	2.3	6.1	0	23	0	0	0	0
	2020	36	100	2.7	2.6	7.5	0	36	0	0	0	0
Wheat flour	2019	4	100	3.2	3.2	5.0	0	4	0	0	0	0
	2020	13	100	3.0	3.3	5.5	0	13	0	0	0	0
Soybean bran	2018	1	100	1.8	1.8	1.8	0	1	0	0	0	0
	2019	3	100	3.2	3.8	4.2	0	3	0	0	0	0
	2020	19	100	4.1	3.4	9.3	0	19	0	0	0	0
Rapeseed meal	2018	24	100	8.5	6.8	14.9	0	16	8	0	0	0
	2019	4	100	7.3	6.1	12.2	0	3	1	0	0	0
	2020	5	100	3.7	3.6	5.6	0	5	0	0	0	0
Peanut meal	2018	27	100	23.1	13.7	59.7	0	9	9	5	4	14.8
	2019	5	100	27.4	32.0	40.7	0	1	1	3	0	0
	2020	9	100	12.7	13.6	21.1	0	4	5	0	0	0
Fish meal	2018	67	100	1.2	1.1	2.5	0	67	0	0	0	0
	2019	12	100	1.2	1.2	1.7	0	12	0	0	0	0
Grass grain	2018	68	100	7.9	6.2	45.7	0	61	4	3	0	4.4
	2019	41	100	4.1	3.8	9.2	0	41	0	0	0	0
	2020	16	100	5.3	5.1	14.0	0	15	1	0	0	0
Unite bran	2018	12	100	6.3	6.3	7.8	0	12	0	0	0	0
	2019	14	100	4.0	3.5	9.2	0	14	0	0	0	0
	2020	15	100	3.4	2.9	11.8	0	14	1	0	0	0
Rice bran	2019	4	100	7.5	5.6	15.3	0	3	1	0	0	0
	2020	14	100	3.7	3.3	7.4	0	14	0	0	0	0
Complete pig feed	2018	317	100	4.9	3.2	59.7	0	295	17	1	4	2.5
	2019	214	100	4.0	2.9	20.9	0	197	17	0	0	0.5
	2020	89	100	3.5	3.0	12.3	0	86	3	0	0	0
Complete poultry feed	2018	248	99.6	4.5	3.4	57.4	1	231	15	0	1	1.2
	2019	144	100	5.7	4.3	31.5	0	127	15	2	0	2.8
	2020	179	100	4.6	4.0	15.6	0	166	13	0	0	0
Complete ruminant feed	2018	117	100	8.5	3.8	77.5	0	99	9	3	6	8.5
	2019	47	100	15.4	10.8	44.3	0	22	15	10	0	40.4
	2020	62	100	4.7	4.2	12.7	0	59	3	0	0	0

^a^Positive samples are defined as those with aflatxoin B_1_ ≥ 0.5 μg/kg (LOD)

### Occurrence of DON in feeds

A total of 3,507 samples, including 2090 feedstuffs and 1,417 complete feeds, were collected during 2018–2020 for DON analysis (Table 3). DON was detected in 96.4–100% of feedstuffs and complete feeds, with the mean values ranging from 458.0–1,925.4 μg/kg. The highest median concentration of DON was 1,529.7 μg/kg found in wheat middling harvested during 2018, followed by 1,449.5 μg/kg in grass grain collected in 2018, 1,370.6–1,381.5 μg/kg in wheat bran harvested during 2018 and 2019, and 1,346.6–1,367.8 μg/kg in dried distillers grains with soluble from 2018 and 2020. The maximum contamination of DON was 9,186.4 μg/kg in wheat middlings harvested in 2018, followed by 6,430.6 μg/kg in dried distillers grains with soluble from 2018, 4,985.2 μg/kg in corn bran from 2018, and 4,505.0 μg/kg in rice bran from 2019. Only 2 samples, 1 wheat middling and 1 dried distillers grains with soluble, were contaminated with DON at concentrations over 5,000 μg/kg. However, 55 complete pig feed samples, which account for 8.9% of all the complete pig feed samples, were contaminated with DON at levels over the Chinese safety standard concentration of 1,000 μg/kg (Table 1).

**Table 3:** Deoxynivalenol concentrations in feeds^a^

Item	Year	NO. of samples	Positive samples, μg/kg				Numbers of samples in the range, μg/kg				The rate of over standard, %
			%	Mean	Medain	Maximum	< 100	100–1000	1000–5000	> 5000	
Corn	2018	229	98.7	574.5	547.3	1,839.2	3	212	14	0	0
	2019	255	99.6	627.1	615.5	1,525.7	1	231	23	0	0
	2020	215	100	686.3	630.8	3,343.6	0	187	28	0	0
Dried distillers grains with soluble	2018	82	100	1,439.2	1,367.8	6,430.6	0	30	51	1	1.2
	2019	22	100	1,171.7	1,057.1	3,004.3	0	9	13	0	0
	2020	23	100	1,570.9	1,346.6	3,343.6	0	5	18	0	0
Corn germ meal	2018	28	100	681.2	649.3	1,741.9	0	26	2	0	0
	2019	23	100	898.8	759.4	2,642.8	0	19	4	0	0
	2020	10	100	1,342.0	908.3	4,039.4	0	5	5	0	0
Corn bran	2018	33	100	1,240.4	939.9	4,985.2	0	19	14	0	0
	2019	19	100	1,093.4	890.2	4,278.4	0	11	8	0	0
	2020	16	100	1,349.2	1,017.7	2,927.9	0	8	8	0	0
Corn gluten meal	2018	21	100	4,58.0	430.7	846.6	0	21	0	0	0
	2019	4	100	504.6	469.8	680.2	0	4	0	0	0
	2020	1	100	741.0	741.0	741.0	0	1	0	0	0
Wheat	2018	110	100	887.6	773.8	2,035.7	0	77	33	0	0
	2019	34	100	775.4	769.6	1,738.2	0	27	7	0	0
	2020	27	100	723.4	678.2	2,790.1	0	25	2	0	0
Wheat middling	2018	34	100	1,925.4	1,529.7	9,186.4	0	4	29	1	2.9
	2019	46	100	983.3	905.6	2,638.7	0	30	16	0	0
	2020	28	96.4	774.6	585.4	2,356.3	1	20	7	0	0
Wheat bran	2018	148	100	1,447.9	1,370.6	1,665.4	0	49	99	0	0
	2019	86	100	1,388.5	1,381.5	3,650.8	0	29	57	0	0
	2020	41	97.6	1,356.1	1,235.3	3,370.5	1	17	23	0	0
Soybean meal	2018	118	98.3	516.9	510.2	967.0	2	116	0	0	0
	2019	23	100	459.6	487.2	659.6	0	23	0	0	0
	2020	36	97.2	530.5	532.5	1,140.6	1	34	1	0	0
Wheat flour	2019	4	100	700.7	698.1	1,151.3	0	3	1	0	0
	2020	13	100	482.1	426.4	855.6	0	13	0	0	0
Soybean bran	2018	1	100	664.0	664.0	664.0	0	1	0	0	0
	2019	4	100	783.7	749.6	1,068.7	0	3	1	0	0
	2020	19	100	1,274.6	1,062.6	2,741.0	0	9	10	0	0
Rapeseed meal	2018	24	100	691.9	622.5	1,321.3	0	20	4	0	0
	2019	4	100	482.7	416.5	785.9	0	4	0	0	0
	2020	5	100	629.7	650.2	701.4	0	5	0	0	0
Peanut meal	2018	27	100	796.3	765.3	1,576.7	0	21	6	0	0
	2019	5	100	1,045.1	1,034.8	1,203.4	0	1	4	0	0
	2020	9	100	603.4	695.4	830.9	0	9	0	0	0
Fish meal	2018	67	100	520.8	469.7	1,082.8	0	66	1	0	0
	2019	12	100	534.2	512.3	956.6	0	12	0	0	0
Grass grain	2018	68	100	1,625.7	1,449.5	4,079.1	0	18	50	0	0
	2019	41	100	1,101.6	968.0	3,712.2	0	22	19	0	0
	2020	16	100	994.5	1,011.1	1,550.5	0	8	8	0	0
Unite bran	2018	12	100	801.5	697.9	1,632.6	0	9	3	0	0
	2019	14	100	672.3	606.2	1,781.3	0	13	1	0	0
	2020	15	100	754.6	676.9	1,240.4	0	9	6	0	0
Rice bran	2019	4	100	1,613.8	744.9	4,505.0	0	3	1	0	0
	2020	14	100	682.1	582.7	1,549.0	0	11	3	0	0
Complete pig feed	2018	317	99.4	572.0	536.8	2,158.6	2	293	22	0	6.9
	2019	214	99.5	744.8	657.1	3,712.2	1	184	29	0	13.6
	2020	89	100	661.0	678.4	1,197.8	0	85	4	0	4.5
Complete poultry feed	2018	248	99.2	539.3	527.0	1,261.5	2	241	5	0	0
	2019	144	100	636.5	542.6	2,638.7	0	124	20	0	0
	2020	180	100	806.6	767.4	2,970.1	0	147	33	0	0
Complete ruminant feed	2018	117	100	640.6	574.3	1,368.1	0	103	14	0	0
	2019	46	97.8	752.2	732.7	2,254.7	1	36	9	0	0
	2020	62	100	863.4	804.2	2,613.7	0	46	16	0	0

^a^Positive samples are defined as those with deoxynivalenol ≥100 μg/kg (LOD)

### Occurrence of ZEN in feeds

A total of 3,499 samples, including 2,089 feedstuffs and 1,415 complete feeds, were collected during 2018–2020 for ZEN analysis (Table 4). ZEN was detected in 96.9–100% of feedstuffs and complete feeds, with the mean concentrations ranging from 48.1–326.8 μg/kg. The highest median value of ZEN was 326.8 μg/kg in corn gluten meal from 2020, followed by 226.0 μg/kg in corn germ meal from 2020, and 168.5 μg/kg in rice bran from 2019. The maximum concentrations of ZEN were 1,599.0 μg/kg found in both grass grain and complete pig feed from 2019, followed by 956.7 μg/kg in dried distillers grains with soluble from 2019, and 906.9 μg/kg in both wheat middlings and complete ruminant feed from 2018 and 2019. A total of 10 feedstuffs and 27 complete feed samples, which account for 0.5% and 1.9% of all the analyzed feedstuffs and complete feed samples, respectively, were contaminated with ZEN at levels over the Chinese safety standard concentration (Table 1).

**Table 4:** Zearalenone concentrations in feeds^a^

Item	Year	NO. of samples	Positive samples, μg/kg				Numbers of samples in the range, μg/kg				The rate of over standard, %
			%	Mean	Medain	Maximum	< 10	10–250	250–500	500–2000	
Corn	2018	229	96.9	68.2	47.3	480.8	7	214	8	0	0
	2019	255	99.6	62.0	48.3	320.0	1	251	3	0	0
	2020	215	100	140.7	108.1	822.0	0	182	28	5	2.3
Dried distillers grains with soluble	2018	82	100	141.1	113.8	614.6	0	72	7	3	0
	2019	22	100	214.0	149.1	956.7	0	13	8	1	0
	2020	23	100	144.9	96.1	350.1	0	18	5	0	0
Corn germ meal	2018	28	100	135.1	64.5	706.7	0	24	2	2	7.1
	2019	23	100	144.9	108.8	416.8	0	18	5	0	0
	2020	10	100	250.1	226.0	561.1	0	5	4	1	10
Corn bran	2018	33	100	146.9	79.7	742.6	0	27	4	2	0
	2019	19	100	105.7	73.7	343.8	0	17	2	0	0
	2020	16	100	183.5	140.6	475.2	0	12	4	0	0
Corn gluten meal	2018	21	100	105.6	69.4	505.7	0	19	1	1	4.8
	2019	4	100	54.9	39.4	116.6	0	4	0	0	0
	2020	1	100	326.8	326.8	326.8	0	0	1	0	0
Wheat	2018	110	99.1	100	83.4	573.7	1	106	2	1	0
	2019	34	97.1	61.2	45.5	210.8	1	33	0	0	0
	2020	27	100	104.9	72.7	369.1	0	24	3	0	0
Wheat middling	2018	34	100	87.7	77.9	190.3	0	34	0	0	0
	2019	45	100	105.9	72.0	906.9	0	42	2	1	0
	2020	28	100	132.9	94.1	852.8	0	26	1	1	0
Wheat bran	2018	148	100	92.0	81.8	280.6	0	146	2	0	0
	2019	85	100	91.5	83.4	346.3	0	83	2	0	0
	2020	41	100	170.6	108.1	604.8	0	32	7	2	0
Soybean meal	2018	118	100	74.3	66.2	339.9	0	116	2	0	0
	2019	23	100	91.1	54.8	522.0	0	21	1	1	0
	2020	36	100	79.7	51.7	288.8	0	34	2	0	0
Wheat flour	2019	4	100	54.0	48.9	87.9	0	4	0	0	0
	2020	13	100	67.5	64.2	207.2	0	13	0	0	0
Soybean bran	2018	1	100	48.1	48.1	48.1	0	1	0	0	0
	2019	4	100	96.3	90.3	176.8	0	4	0	0	0
	2020	19	100	177.7	113.5	826.8	0	14	5	0	0
Rapeseed meal	2018	24	100	79.9	66.2	336.3	0	23	1	0	0
	2019	4	100	59.3	50.9	92.1	0	4	0	0	0
	2020	5	100	58.3	47.8	85.3	0	5	0	0	0
Peanut meal	2018	27	100	76.1	76.9	118.1	0	27	0	0	0
	2019	5	100	89.4	79.3	118.1	0	5	0	0	0
	2020	9	100	105.8	77.3	227.9	0	9	0	0	0
Fish meal	2018	67	100	54.3	48.8	175.9	0	67	0	0	0
	2019	12	100	50.2	31.3	175.9	0	12	0	0	0
Grass grain	2018	66	100	115.9	88.2	614.6	0	62	2	2	0
	2019	40	100	125.4	74.3	1,599.0	0	38	1	1	2.5
	2020	16	100	206.1	140.8	674.2	0	11	4	1	0
Unite bran	2018	12	100	60.8	45.9	127.2	0	12	0	0	0
	2019	13	100	117.2	66.5	478.1	0	12	1	0	0
	2020	15	100	109.5	95.9	278.7	0	14	1	0	0
Rice bran	2019	4	100	176.9	168.5	299.3	0	3	1	0	0
	2020	14	100	75.8	62.3	278.7	0	13	1	0	0
Complete pig feed	2018	317	98.4	67.2	48.1	513.0	5	301	9	2	3.5
	2019	213	100	84.3	65.6	1,599.0	0	209	2	2	1.9
	2020	89	100	93.1	91.6	268.1	0	88	1	0	3.4
Complete poultry feed	2018	248	99.6	59.8	51.5	331.9	1	245	2	0	0
	2019	144	100	109.2	75.2	622.4	0	133	8	3	2.1
	2020	179	100	155.1	118.8	852.8	0	153	22	4	2.2
Complete ruminant feed	2018	117	100	90.6	50.3	906.9	0	107	8	2	1.7
	2019	46	97.8	79.2	77.5	223.1	1	45	0	0	0
	2020	62	100	124.9	105.5	376.3	0	55	7	0	0

^a^Positive samples are defined as those with zearalenone ≥10 μg/kg (LOD)

### Co-occurrence of AFB_1_, DON and ZEN in feeds

The co-occurrence of AFB_1_, DON and ZEN in feedstuffs and complete feed samples during 2018–2020 were presented in Table 5. The co–occurrence of AFB_1_ + DON, AFB_1_ + ZEN, DON+ZEN, and AFB_1_ + DON+ZEN in feed ingredients ranged from 81.9–100%, 81.5–100%, 96.1–100% and 81.5–100%, respectively. Notably, the co-contaminates of AFB_1_ + DON, AFB_1_ + ZEN, DON+ZEN, along with AFB_1_ + DON+ZEN in complete feeds ranged from 97.8–100%, 97.8–100%, 95.7–100% and 95.7–100%, respectively.

**Table 5:** Percentage of AFB_1_, DON and ZEN co-occurrence in feeds^a^

Item	Year	AFB_1_ + DON, %	AFB_1_ + ZEN, %	DON+ZEN, %	AFB_1_ + DON+ZEN, %
Corn	2018	94.8	93.4	96.1	93.0
	2019	81.9	81.5	99.2	81.5
	2020	98.1	98.1	100	98.1
Wheat	2018	99.1	98.2	99.1	98.2
	2019	97.1	94.1	97.1	94.1
	2020	100	100	100	100
Wheat middling	2018	100	100	100	100
	2019	100	100	100	100
	2020	96.4	100	96.4	96.4
Wheat bran	2018	100	100	100	100
	2019	100	100	100	100
	2020	100	100	100	100
Soybean meal	2018	98.3	100	98.3	98.3
	2019	100	100	100	100
	2020	97.2	100	97.2	97.2
Soybean bran	2018	100	100	100	100
	2019	100	100	100	100
	2020	100	100	100	100
Corn bran	2018	100	100	100	100
	2019	100	100	100	100
	2020	100	100	100	100
Corn gluten meal	2018	100	100	100	100
	2019	100	100	100	100
	2020	100	100	100	100
Corn germ meal	2018	100	100	100	100
	2019	100	100	100	100
	2020	100	100	100	100
Unite bran	2018	100	100	100	100
	2019	100	100	100	100
	2020	100	100	100	100
Rapeseed meal	2018	100	100	100	100
	2019	100	100	100	100
	2020	100	100	100	100
Peanut meal	2018	100	100	100	100
	2019	100	100	100	100
	2020	100	100	100	100
Dried distillers grains with soluble	2018	100	100	100	100
	2019	100	100	100	100
	2020	95.7	95.7	100	95.7
Grass grain	2018	100	100	100	100
	2019	100	100	100	100
	2020	100	100	100	100
Fish meal	2018	100	100	100	100
	2019	100	100	100	100
Wheat flour	2019	100	100	100	100
	2020	100	100	100	100
Rice bran	2019	100	100	100	100
	2020	100	100	100	100
Complete pig feed	2018	99.4	98.4	97.8	97.8
	2019	99.5	100	96.7	99.5
	2020	100	100	100	100
Complete poultry feed	2018	99.2	99.2	98.8	98.4
	2019	100	100	100	100
	2020	100	100	100	100
Complete ruminant feed	2018	100	100	100	100
	2019	97.8	97.8	95.7	95.7
	2020	100	100	100	100

^a^*AFB*_*1*_ aflatxoin B_1_; *DON* deoxynivalenol; *ZEN* zearalenone; *AFB*_*1*_ *+ DON* feeds co-contaminated with AFB_1_ and DON; *AFB*_*1*_ *+ ZEN* feeds co-contaminated with AFB_1_ and ZEN; DON+ZEN, feeds co-contaminated with DON and ZEN; AFB_1_ + DON+ZEN, feed co-contaminated with AFB_1_, DON and ZEN

## Discussion

The present study was carried out to investigate the individual and combined contamination of the most prevalent and toxic mycotoxins, AFB_1_, DON and ZEN, in feedstuffs and complete feeds harvested from various regions of China between 2018 and 2020. In general, the three analyzed mycotoxins displayed a considerably high occurrence in the analyzed feed samples, ranging from 81.9–100%, 96.4–100%, and 96.9–100% for AFB_1_, DON and ZEN, respectively. The average concentration of AFB_1_ (1.2–27.4 μg/kg) determined in this study was lower than formerly reported concentrations (0.4–627 μg/kg) from samples harvested between 2013 and 2015 in China [21, 22], while higher than concentrations (1.6–10.0 μg/kg) from samples harvested between 2016 and 2017 in China [4]. Although only 0.9% of the analyzed raw feed ingredients (corn, corn bran, wheat middling, wheat bran, peanut meal, and grass grain) with AFB_1_ exceeded the Chinese safety standard concentration, 1.5%, 1.2% and 12.8% of all the analyzed final products for pig, poultry and ruminant contained AFB_1_ over the limitation of Chinese safety standard. These results are much higher than the previously reported that 1.0% analyzed feed samples with AFB_1_ exceeded China’s safety standards [4]. These divergences could be due to the fact that the analyzed feed samples were randomly gathered from different regions, and weather varies in these areas during the harvest period. Owing to AFB_1_ is the most toxic mycotoxin [6, 28, 29], it is important to persist in supervising the concentration of AFB_1_ in the raw feed ingredients and final products in the future.

The occurrence and level of DON in the analyzed feed samples in this study were quite high. The percentage of positive samples of DON was 96.4–100%, which is higher than the previously reported 50.0–100% in feeds collected in China during 2013–2017 [4, 21, 22]. The average concentration of DON in feeds ranged between 458.0–1,925.4 μg/kg, which is relatively lower than the previously reported range of 364.5–4,381.5 μg/kg in the feeds collected in China between 2013 and 2017 [4, 21, 22]. Although only 0.1% of analyzed feed ingredients contaminated with DON exceeded China’s safety standards, 8.9% of the complete pig feed samples that were contaminated with DON over the limitation of the safety standards of China. These findings remind us that we need to be cognizant of the potential for contamination of the raw feed ingredients, including corn bran, dried distillers grains with soluble, wheat middling, wheat bran, and grass grain, which were relatively severely contaminated by DON with an average concentrations more than 1,000 μg/kg.

The occurrence of ZEN (96.9–100%) in the analyzed feed samples in the current study was higher than the previously reported (50.0–100%) from harvests between 2013 and 2017 [4, 21, 22]. However, the concentration of ZEN (48.1–326.8 μg/kg) in the analyzed feed samples was relatively lower in this study than the previously reported (0–729.2 μg/kg) from harvests between 2013 and 2017 [4, 21, 22]. These differences could be due to the various sampling regions and different weather conditions during the harvest periods. Notably, 0.5% of all the analyzed feedstuff samples, including corn, corn gluten meal, corn germ meal and grass grain, were contaminated with ZEN at concentrations that exceeded the Chinese safety standard level. Meanwhile, 2.9%, 1.2% and 0.9% of all the analyzed complete feeds for pig, poultry and ruminant contained ZEN that exceeded the regulatory limits in China; this finding was much lower than previously reported, whereby 10.7% of the complete pig feeds were shown to be contaminated with ZEN exceeding the regulatory limits [4].

Mycotoxins co-contamination can exert additive and synergistic toxic effects, which have been well-documented [3, 30–34]. Unfortunately, co-contamination of mycotoxins in feeds was extremely universal in this study, with more than 81.5% of feed samples containing 2 or more mycotoxins. Notably, corn bran, corn gluten meal, corn germ meal, wheat bran, wheat flour, unite bran, rice bran, soybean bran, rapeseed meal, peanut meal, fish meal and grass grain were 100% co-contaminated with AFB_1_, DON and ZEN. Meanwhile, more than 97.8%, 98.4% and 95.7% complete feeds for pig, poultry and ruminant, respectively, were also co-contaminated with these three mycotoxins. These results were similar to previous reports which showed that mycotoxin co-contamination is a widespread issue in the feed industry [21, 35–38]. Since the present feed safety regulations do not consider the potential toxicity of co-contamination of mycotoxins, their combined toxicity on animal health and production may be underestimated, and the combined toxicity of these mycotoxins warrants further study so that it might be considered when new regulatory limits for mycotoxins are set in the future.

It is also worth noting that the average concentrations of AFB_1_ and DON were not different in the analyzed feeds amongst the three harvest years, while the mean levels of ZEN were much higher in most of the feedstuffs and all the complete feeds in the year 2020 in comparison to years 2018 and 2019. Meanwhile, the raw feed ingredients, corn, dried distillers grains with soluble, corn gluten meal, corn germ meal, corn bran, wheat middling, wheat bran, peanut meal, and grass grain, were seriously contaminated with more than one mycotoxin. Thus, these ingredients need to be regularly monitored. Moreover, strategies for the control of mycotoxins are needed to be seriously considered. Generally, during the pre-harvest, good field and storage management strategies, including crop rotation, variety choice, use of fungicide and antagonistic fungi, temperature, moisture content, humidity of the environment, are important to prevent the mycotoxigenic fungal development and mycotoxin formation [39, 40]. During the post-harvest, physical, chemical and biological approaches have been used to decontaminate mycotoxins from the feedstuffs [39, 40]. So far, the application of binders’ adsorption of mycotoxins from the gastrointestinal tract of animals is the most effective way in practice [40, 41]. While development of novel microorganisms or their enzymes used to biodegradation of the mycotoxins is also a promising approach [39, 40, 42].

## Conclusion

In conclusion, this study found that AFB_1_, DON and ZEN were highly prevalent in all the analyzed feed samples collected from different areas of China between 2018 and 2020. Notably, 0.9%, 0.5% and 0.1% of analyzed raw feed ingredients exceeded China’s safety standards for AFB_1_, ZEN and DON, respectively. However, much higher ratios of AFB_1_ (1.2–12.8%), ZEN (0.9–2.9%) and DON (0–8.9%) in complete feeds for pigs, poultry and ruminant exceeded China’s safety standards. Moreover, the co-contamination of AFB_1_, DON and ZEN was quite common in both the raw feed ingredients (81.5–100%) and complete feed products (95.7–100%). Taken together, these outcomes remind us that, 1) contamination of mycotoxins in feeds needs to be regularly monitored, 2) suitable remediation strategies for mycotoxins need to be applied in the feed industry, and 3) new regulatory limits should consider mycotoxin co-contamination in the feeds.

## Data Availability

The datasets used and/or analyzed during the current study are publicly available.

## Abbreviations

AFB_1_: Aflatoxin B_1_
DON: Deoxynivalenol
ZEN: Zearalenone
HPLC: High-performance liquid chromatography
LOD: Limits of detection
LOQ: Limits of quantification.
